# Toxic epidermal necrolysis induced by axitinib in a patient with advanced lung adenocarcinoma

**DOI:** 10.1093/skinhd/vzae028

**Published:** 2025-02-14

**Authors:** Na Wang, Dongkai Li, Huaiwu He, Yun Long, Dawei Liu

**Affiliations:** Department of Critical Care Medicine, State Key Laboratory of Complex Severe and Rare Diseases, Peking Union Medical College Hospital, Chinese Academy of Medical Sciences, Beijing, China; Department of Critical Care Medicine, State Key Laboratory of Complex Severe and Rare Diseases, Peking Union Medical College Hospital, Chinese Academy of Medical Sciences, Beijing, China; Department of Critical Care Medicine, State Key Laboratory of Complex Severe and Rare Diseases, Peking Union Medical College Hospital, Chinese Academy of Medical Sciences, Beijing, China; Department of Critical Care Medicine, State Key Laboratory of Complex Severe and Rare Diseases, Peking Union Medical College Hospital, Chinese Academy of Medical Sciences, Beijing, China; Department of Critical Care Medicine, State Key Laboratory of Complex Severe and Rare Diseases, Peking Union Medical College Hospital, Chinese Academy of Medical Sciences, Beijing, China

## Abstract

Patients taking oral EGFR inhibitors should be alert to the risk of TEN if they suddenly develop widespread rash.

Dear Editor, A 71-year-old Chinese man was admitted to our intensive care unit in June 2023 due to a severe drug eruption. He was diagnosed with right lung adenocarcinoma accompanied by bone, meningeal and supraclavicular lymph node metastasis (T2aN2M1b, IVb). Next-generation sequencing revealed the presence of an *EGFR* mutation in exon 21 (c.2573T>G, p.L858R) and exon 19 (c.2232C>G, p.I1744M). He then received axitinib 80 mg daily. Due to refractory dizziness and double vision after 1 month, the dose was increased to 160 mg daily. After 35 days, the patient suddenly developed high fever and diffused skin lesions characterized by erythema on the trunk, four limbs, head and face. Despite discontinuing axitinib, the erythema rapidly progressed into bullae formation along with dermolysis and exfoliation extending from the back to the thighs (Figure 1), and Nikolski sign was positive. The involved skin lesions exceeded 30% of the body surface area (BSA). Upon admission, assessment using SCORTEN (SCORe for Toxic Epidermal Necrolysis) criteria which includes age >40 years old (+1 point), presence of malignant tumours (+1 point) and epidermal detachment >10% BSA (+1 point). Three points were assigned for this patient’s condition. Simultaneously, the patient developed oral ulcer and conjunctivitis. An ophthalmic examination demonstrated diffuse congestion of bulbar and palpebral conjunctivas. All bacteriological cultures conducted on blood samples, as well as urine and sputum specimens, yielded negative results. Given these findings and a history of exposure to axitinib, toxic epidermal necrolysis (TEN) was clinically diagnosed. Initially, the patient received intravenous methylprednisolone at a dose of 1.8 mg kg^-1^ daily for 5 days, followed by a reduced dose of 0.9 mg kg^-1^ daily for 3 days. Subsequently, oral prednisolone was initiated at a daily dose of 40 mg and gradually tapered by reducing the dose by 10 mg every 2 days until discontinuation. Concurrently, regular treatment with colobital and ciclosporin eye drops was administered. Following a stay in the intensive care unit of 10 days and dermatology management spanning 20 days, there was gradual improvement in the extent of skin desquamation (Figure 1).

**Figure 1:**
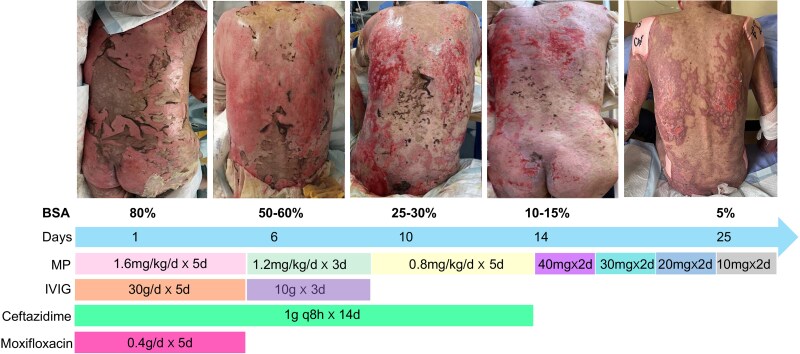
An overview of skin lesions changing according to the therapeutic agents used. BSA, body surface area; IVIG, intravenous immunoglobulin; MP, methylprednisolone.

TEN is a drug-induced hypersensitivity reaction commonly triggered by medications such as nonsteroidal anti-inflammatory agents and antibiotics.^1^ The incidence of immune checkpoint inhibitor (ICI)-induced TEN ranges from approximately 2% to 3%; however, the mortality rate from ICI-induced TEN is nearly 71%.^2^ Reported ICIs that trigger TEN primarily encompass anti-cytotoxic T lymphocyte ­antigen 4 antibodies, as well as anti-programmed cell death protein 1/anti-programmed death ligand 1.^3^ In this particular case, TEN was triggered by axitinib, an epidermal growth factor receptor (EGFR) reporter inhibitor, which is relatively rare. ICI-induced TEN directly activates cytotoxic T cells, leading to apoptosis of normal keratinocytes in TEN.^4^ Clinically, ICI-induced TEN presents atypically with delayed onset (up to 12 weeks) and a high mortality rate.^4^ ICIs have prolonged half-lives (e.g. axitinib T1/2 = 2 days) resulting in persistent mucocutaneous toxicity even after discontinuation of ICIs for >10 days. Like other severe immune-related adverse events, systemic corticosteroid administration is recommended for the treatment of ICI-induced TEN. In our case, intravenous methylprednisolone was administered at a dose of 1.8–0.8 mg kg^-1^ daily for 2 weeks, leading to gradual reduction in skin lesion area (from 80% to 15% affected BSA) and complete healing of the corneal ulcer (Figure 1). Subsequently, the patient’s medication was switched from methylprednisolone to oral prednisone until discontinuation, leading to successful discharge from hospital. Finally, furmonertinib treatment was administered without any observed side-effects. Furmonertinib, characterized by its high selectivity and safety profile as a third-generation EGFR–tyrosine kinase inhibitor (TKI), exhibits minimal adverse events compared with other EGFR–TKIs, and the incidence rate of erythema is only 7%.^5^ Therefore, furmonertinib may be a potential treatment strategy and requires further clinical evaluation.

## Data Availability

The data underlying this article are available in the article.
